# Network analysis of long non-coding RNA expression profiles in common warts

**DOI:** 10.1016/j.heliyon.2022.e11790

**Published:** 2022-11-19

**Authors:** Amneh H. Tarkhan, Laith N. AL-Eitan, Rami Q. Alkhatib, Mansour A. Alghamdi

**Affiliations:** a Department of Biotechnology and Genetic Engineering, College of Science and Arts, Jordan University of Science and Technology, Irbid, 22110, Jordan; b Department of Anatomy, College of Medicine, King Khalid University, Abha, 61421, Saudi Arabia; c Genomics and Personalized Medicine Unit, College of Medicine, King Khalid University, Abha, 61421, Saudi Arabia

**Keywords:** Warts, Human papillomavirus, lncRNA, *CERNA2*, *LINC02159*, *SH3PXD2A-AS1*, *UNC5B-AS1*

## Abstract

**Background:**

Long non-coding RNAs (lncRNAs) have been the subject of considerable attention in recent years due to their role in gene regulation. However, the function of lncRNAs remains poorly understood, especially in the context of infection with low-risk human papillomaviruses (HPVs). To further understanding on this issue, we investigated lncRNA expression in HPV-induced common warts.

**Methods:**

A publicly available high-throughput sequencing dataset for common warts was downloaded from the Gene Expression Omnibus (GEO). lncRNA profiles were generated using the NetworkAnalyst 3.0 workflow, and a list of differentially expressed (DE) lncRNAs in common warts was identified and inputted into the ENCODE, RegNetwork, DisGeNet, and miRNet platforms.

**Results:**

A total of 54 lncRNAs were revealed to be significantly dysregulated in common warts. Of these 54 lncRNAs, 24 and 30 were upregulated and downregulated, respectively. The most significantly differentially expressed lncRNAs in common warts included the *CERNA2*, *LINC02159*, *SH3PXD2A-AS1*, and *UNC5B-AS1* genes.

**Conclusion:**

The current findings suggest that HPV-induced warts impact the host lncRNA transcriptome. To the best of our knowledge, the present study is the first to explore the impact of low-risk HPV infection on lncRNA expression profiles.

## 1 Introduction

Long non-coding RNAs (lncRNAs) are a heterogeneous group of non-protein-coding RNA transcripts that are greater than 200 nucleotides in length and which function in many biological processes as well as diseases [1]. The function of the majority of identified lncRNAs in humans remains to be elucidated, but it is clear that lncRNAs are involved in the regulation of gene expression [2, 3]. lncRNA dysregulation has been associated with various cancers in humans and can be induced in response to infection [4]. Viral infection can also influence the host genome to express lncRNAs that are essential for viral pathogenesis [5, 6]. For example, human papillomavirus (HPV) infection has been reported to widely alter the lncRNA transcriptome of its host [7].

The HPVs are a family of non-enveloped DNA viruses with a preferential tropism for epithelial tissue [8]. More specifically, HPVs infect keratinocytes, the most common epidermal cells, and their replication cycle is tightly linked to keratinocyte differentiation [9]. With over 200 types identified, HPVs are divided into high-risk and low-risk groups based on their oncogenic potential [10]. In immunocompetent individuals, low-risk HPV infection is self-limiting and primarily manifests in the form of benign hyper-proliferative epithelial lesions known as warts [10]. Wart formation typically entails the enlargement of the epithelial cells, thickening of the epithelium, folding of the basal epithelial layer, and cornification of the outer epithelial layer [11]. The most prevalent type of cutaneous wart is the common wart (*Verruca vulgaris*), which accounts for 70% of warts and is preferentially located on the hands [12].

Despite affecting nearly 1 in 10 people, warts have not been the subject of much genetic research, and current treatments mostly rely on damaging or destroying the infected epithelia [13, 14]. In addition, little is known about how lncRNA expression profiles are impacted by low-risk HPV infection. Therefore, the aim of this study was to explore how HPV-induced common warts modulated host expression of lncRNAs.

## 2 Methods

### 2.1 Data acquisition

The dataset investigated in the present study was obtained from The National Center for Biotechnology Information's (NCBI) Gene Expression Omnibus (GEO) repository (accession number GSE136347). The data contained paired epidermal tissue samples from warts (n = 12) and normal skin (n = 12) that were profiled on the Illumina HiSeq 2500 platform. According to the original study, total RNA was extracted from the samples using an RNeasy Mini Kit (Qiagen, Germany), and the purity of the samples was assessed using the Agilent Bioanalyzer (Agilent, USA) [15]. All the samples analyzed in the present study were obtained from Jordanian Arab males.

### 2.2 Data processing

Quality control, filtering, normalization, differential expression analysis, and data visualization were carried out according to the NetworkAnalyst 3.0 workflow [16, 17, 18, 19, 20]. Quality control was performed to check the distribution of raw read counts, the sum of read counts for each sample, similarities and differences between the samples, and the relative distribution of different counts in each group.

Both variance and abundance filtering were applied to the dataset. The 15^th^ percentile of genes with the lowest variance (whose expression values did not change across the samples) and the 4^th^ percentile of genes with the lowest abundance were removed. Based on this filtering step, 37 genes with low variance and 0 genes with low abundance were excluded from downstream analysis.

**Figure 1:**
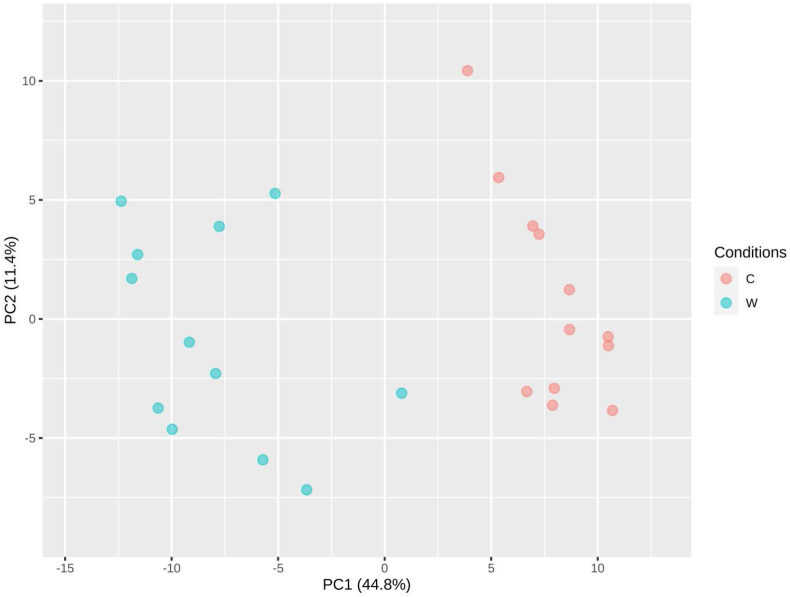
Principal component analysis (PCA) plot of the sample coordinates.

The data was uploaded to the Network Analyst 3.0 web platform and normalized via log2 transformation. The normalized data was displayed in a principal component analysis (PCA) plot and visually inspected for outliers (Figure 1). Differential expression was identified and quantified using EdgeR, where a lncRNA's expression was deemed significant if it had an adjusted p-value of <0.05, resulting in 229 differentially expressed (DE) lncRNAs. Upon applying a cutoff of log2 fold change of >1 (equal to a fold change of 2 on the original scale), a total of 54 lncRNAs were identified as significant.

### 2.3 Integrative pathway analysis

Gene ontology (GO) analysis was performed as part of the NetworkAnalyst 3.0 workflow to determine which biological processes, cellular components, and molecular functions were enriched in common warts.

### 2.4 Interaction network analysis

#### 2.4.1 Using data from integrated knowledge bases

As part of the NetworkAnalyst 3.0 workflow, the list of DE lncRNAs were inputted into the ENCODE, RegNetwork, DisGeNet, and miRNet platforms. ENCODE was used to obtain information about interactions between transcription factors and gene targets, while coregulatory interaction data was obtained from RegNetwork. The DisGeNet database was used to determine lncRNA-disease association data, and miRNet was used to predict the interactions between DE lncRNAs and miRNA targets [21, 22, 23].

#### 2.4.2 Using data from previously published studies

The interaction between DE lncRNA, DE miRNA and DE genes in common warts was carried out using miRNet 2.0, a network-based visual analytic tool for miRNA functional analysis [24]. A total of 54 DE lncRNA, 6 DE miRNA [25], and 3,140 DE genes [15] were uploaded to miRNet 2.0 and submitted for analysis.

### 2.5 Validation of the differentially expressed lncRNA genes

A list of the most differentially methylated (DM) genes [26] in common warts from a previous study was merged with the significantly DE lncRNA genes. Overlapping genes, i.e., those that had significant levels of differential expression and methylation, were considered to be validated.

## 3 Results

### 3.1 Identification of differentially expressed genes

Table 1 shows the 54 lncRNAs that were significantly DE (logFC>1 and adj. p-value<0.05) in common warts. The average GeneCards Inferred Functionality Score (GIFtS) for these lncRNA genes was 15 out of 100, indicating a low level of knowledge about their functionality.

**Table 1:** Significantly differentially expressed lncRNAs in common warts (log2FC > 1 and adj. p-value<0.05) as sorted by the likelihood ratio (LR) test.

Gene ID	Gene	Gene description	Chr	Regulation	GIFtS	LR	logFC	logCPM	P-value	Adj. P-value
642934	CERNA2	competing endogenous lncRNA 2 for microRNA let-7b	10	Down	11	260.13	-4.4329	9.821	1.61 × 10^−58^	3.69 × 10^−56^
285629	LINC02159	long intergenic non-protein coding RNA 2159	5	Down	9	197.18	-3.5974	10.422	8.60 × 10^−45^	9.85 × 10^−43^
100505839	SH3PXD2A-AS1	SH3PXD2A antisense RNA 1	10	Down	11	127.34	-2.2645	12.518	1.57 × 10^−29^	1.20 × 10^−27^
728978	UNC5B-AS1	UNC5B antisense RNA 1	10	Down	12	93.272	-5.6904	10.919	4.56 × 10^−22^	2.61 × 10^−20^
101930071	LOC101930071	uncharacterized LOC101930071	19	Down	9	81.138	-3.1654	10.051	2.11 × 10^−19^	9.64 × 10^−18^
100134229	KDM7A-DT	KDM7A divergent transcript	7	Down	11	72.383	-2.3134	12.95	1.77 × 10^−17^	6.76 × 10^−16^
283404	LINC00592	long intergenic non-protein coding RNA 592	12	Down	14	71.189	-2.267	10.78	3.25 × 10^−17^	1.06 × 10^−15^
400043	LINC02381	long intergenic non-protein coding RNA 2381	12	Up	9	67.221	2.1658	9.4481	2.43 × 10^−16^	6.95 × 10^−15^
100506668	NRAV	negative regulator of antiviral response	12	Down	13	64.243	-1.5483	11.367	1.10 × 10^−15^	2.80 × 10^−14^
100505783	OSER1-DT	OSER1 divergent transcript	20	Up	10	64.005	1.7721	9.4538	1.24 × 10^−15^	2.84 × 10^−14^
283487	LINC00346	long intergenic non-protein coding RNA 346	13	Down	18	49.905	-1.9987	9.3493	1.61 × 10^−12^	3.36 × 10^−11^
541471	MIR4435-2HG	MIR4435-2 host gene	2	Down	16	47.099	-2.6717	9.5312	6.75 × 10^−12^	1.20 × 10^−10^
102723775	LINC01315	long intergenic non-protein coding RNA 1315	22	Up	13	47.084	2.0327	9.7806	6.80 × 10^−12^	1.20 × 10^−10^
678655	CD27-AS1	CD27 antisense RNA 1	12	Up	15	43.799	1.3588	10.83	3.64 × 10^−11^	5.95 × 10^−10^
100507316	MINCR	MYC-induced long non-coding RNA	8	Up	12	41.933	1.3626	9.0001	9.45 × 10^−11^	1.44 × 10^−09^
255480	TBX5-AS1	TBX5 antisense RNA 1	12	Up	16	40.757	3.109	9.8233	1.72 × 10^−10^	2.47 × 10^−09^
101928100	LOC101928100	uncharacterized LOC101928100	12	Down	9	40.003	-1.9292	9.059	2.54 × 10^−10^	3.23 × 10^−09^
100127888	SLCO4A1-AS1	SLCO4A1 antisense RNA 1	20	Up	13	39.492	1.4492	10.405	3.29 × 10^−10^	3.97 × 10^−09^
728431	LINC01137	long intergenic non-protein coding RNA 1137	1	Down	13	37.905	-1.4187	11.64	7.43 × 10^−10^	8.50 × 10^−09^
100505881	MAGI2-AS3	MAGI2 antisense RNA 3	7	Up	16	35.292	1.4344	9.7449	2.84 × 10^−09^	3.09 × 10^−08^
101927402	LOC101927402	uncharacterized LOC101927402	2	Down	n/a	34.75	-1.5469	8.683	3.75 × 10^−09^	3.90 × 10^−08^
401264	TRAM2-AS1	TRAM2 antisense RNA 1 (head-to-head)	6	Up	12	34.665	1.2705	9.0494	3.92 × 10^−09^	3.90 × 10^−08^
399959	MIR100HG	mir-100-let-7a-2-mir-125b-1 cluster host gene	11	Up	17	33.34	2.6187	9.6896	7.74 × 10^−09^	7.38 × 10^−08^
100507602	TRIM52-AS1	TRIM52 antisense RNA 1 (head-to-head)	5	Up	15	33.095	1.3968	10.138	8.78 × 10^−09^	8.04 × 10^−08^
100996579	LINC01806	long intergenic non-protein coding RNA 1806	2	Down	11	32.066	-1.6645	9.9762	1.49 × 10^−08^	1.31 × 10^−07^
29994	BAZ2B	bromodomain adjacent to zinc finger domain 2B	2	Down	39	29.647	-1.0094	11.866	5.18 × 10^−08^	4.40 × 10^−07^
100652740	PYCARD-AS1	PYCARD antisense RNA 1	16	Down	13	29.379	-1.6637	9.1733	5.95 × 10^−08^	4.74 × 10^−07^
25786	DGCR11	DiGeorge syndrome critical region gene 11	22	Down	17	29.361	-1.1633	9.6221	6.01 × 10^−08^	4.74 × 10^−07^
101927164	LOC101927164	uncharacterized LOC101927164	1	Up	9	28.281	1.1633	11.945	1.05 × 10^−07^	7.75 × 10^−07^
100131193	CCDC183-AS1	CCDC183 antisense RNA 1	9	Down	13	27.19	-1.3601	10.044	1.84 × 10^−07^	1.32 × 10^−06^
407975	MIR17HG	miR-17-92a-1 cluster host gene	13	Down	26	26.62	-1.4434	8.8305	2.48 × 10^−07^	1.72 × 10^−06^
283932	FBXL19-AS1	FBXL19 antisense RNA 1	16	Down	18	26.506	-1.3202	8.7731	2.63 × 10^−07^	1.77 × 10^−06^
100506233	RAB30-DT	RAB30 divergent transcript	11	Down	10	24.585	-1.1745	9.2704	7.11 × 10^−07^	4.65 × 10^−06^
55384	MEG3	maternally expressed 3	14	Up	30	23.383	2.1667	11.701	1.33 × 10^−06^	8.45 × 10^−06^
101927989	LOC101927989	uncharacterized LOC101927989	18	Down	n/a	21.552	-1.046	8.4506	3.44 × 10^−06^	2.13 × 10^−05^
400236	FOXN3-AS1	FOXN3 antisense RNA 1	14	Down	13	21.362	-1.2758	8.8987	3.80 × 10^−06^	2.29 × 10^−05^
339400	FLG-AS1	FLG antisense RNA 1	1	Down	16	20.097	-1.4624	9.7498	7.36 × 10^−06^	4.21 × 10^−05^
100507463	PSMB8-AS1	PSMB8 antisense RNA 1 (head-to-head)	6	Up	12	19.217	1.1362	9.6749	1.17 × 10^−05^	6.36 × 10^−05^
10866	HCP5	HLA complex P5	6	Up	29	17.185	1.138	12.502	3.39 × 10^−05^	1.69 × 10^−04^
100131213	ZNF503-AS2	ZNF503 antisense RNA 2	10	Down	19	16.92	-1.1135	9.3593	3.90 × 10^−05^	1.90 × 10^−04^
112597	CYTOR	cytoskeleton regulator RNA	2	Down	18	16.512	-1.2746	8.7245	4.83 × 10^−05^	2.27 × 10^−04^
100506930	LINC00665	long intergenic non-protein coding RNA 665	19	Up	15	16.289	1.4209	9.1863	5.44 × 10^−05^	2.49 × 10^−04^
100131096	TNRC6C-AS1	TNRC6C antisense RNA 1	17	Up	9	16.084	1.4816	9.4413	6.06 × 10^−05^	2.69 × 10^−04^
26220	DGCR5	DiGeorge syndrome critical region gene 5	22	Up	20	15.747	1.1055	9.0953	7.24 × 10^−05^	2.96 × 10^−04^
283335	LOC283335	uncharacterized LOC283335	12	Up	12	15.667	1.2341	8.4712	7.55 × 10^−05^	3.04 × 10^−04^
100128398	LOC100128398	uncharacterized LOC100128398	19	Up	12	14.59	1.0858	8.2482	1.34 × 10^−04^	4.93 × 10^−04^
100505635	DAAM2-AS1	DAAM2 antisense RNA 1	6	Down	10	14.36	-1.2434	8.7449	1.51 × 10^−04^	5.32 × 10^−04^
100124700	HOTAIR	HOX transcript antisense RNA	12	Up	26	13.843	1.1986	11.077	1.99 × 10^−04^	6.89 × 10^−04^
378938	MALAT1	metastasis associated lung adenocarcinoma transcript 1	11	Down	23	13.175	-1.0917	17.296	2.84 × 10^−04^	9.55 × 10^−04^
101929072	MICA-AS1	MICA antisense RNA 1	6	Up	8	12.223	1.0197	8.7416	4.72 × 10^−04^	1.51 × 10^−03^
285084	LINC01305	long intergenic non-protein coding RNA 1305	2	Up	11	11.252	1.1378	9.1403	7.95 × 10^−04^	2.22 × 10^−03^
102724532	LOC102724532	uncharacterized LOC102724532	17	Down	n/a	10.645	-1.0192	10.996	1.10 × 10^−03^	2.90 × 10^−03^
503538	A1BG-AS1	A1BG antisense RNA 1	19	Up	13	10.055	1.1061	8.3948	1.52 × 10^−03^	3.77 × 10^−03^
129790	LINC01006	long intergenic non-protein coding RNA 1006	7	Down	20	9.975	-1.009	9.0021	1.59 × 10^−03^	3.87 × 10^−03^

*Adj. p-value*: adjusted p-value; *Chr*: chromosome; *GIFtS*: GeneCards Inferred Functionality Score; *logCPM*: base 2 logarithm of counts per million; *logFC*: base 2 logarithm of fold change; *LR*: likelihood ratio.

**Figure 2:**
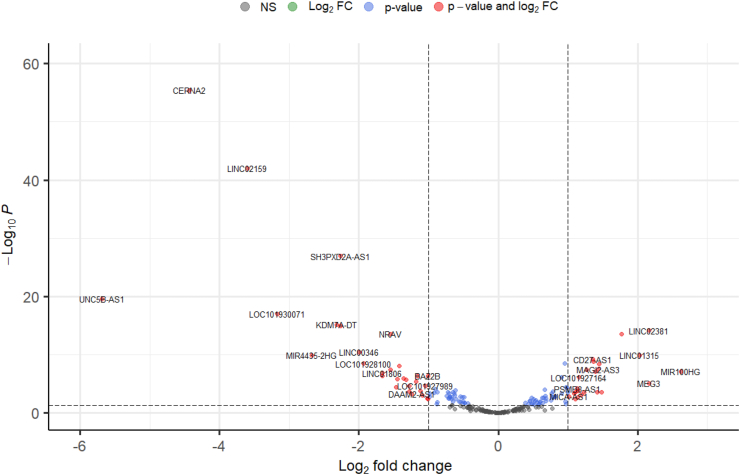
Volcano plot of lncRNA expression in common warts. *Upregulated lncRNA are red, downregulated lncRNA are blue, and non-significant lncRNA are grey.*

**Figure 3:**
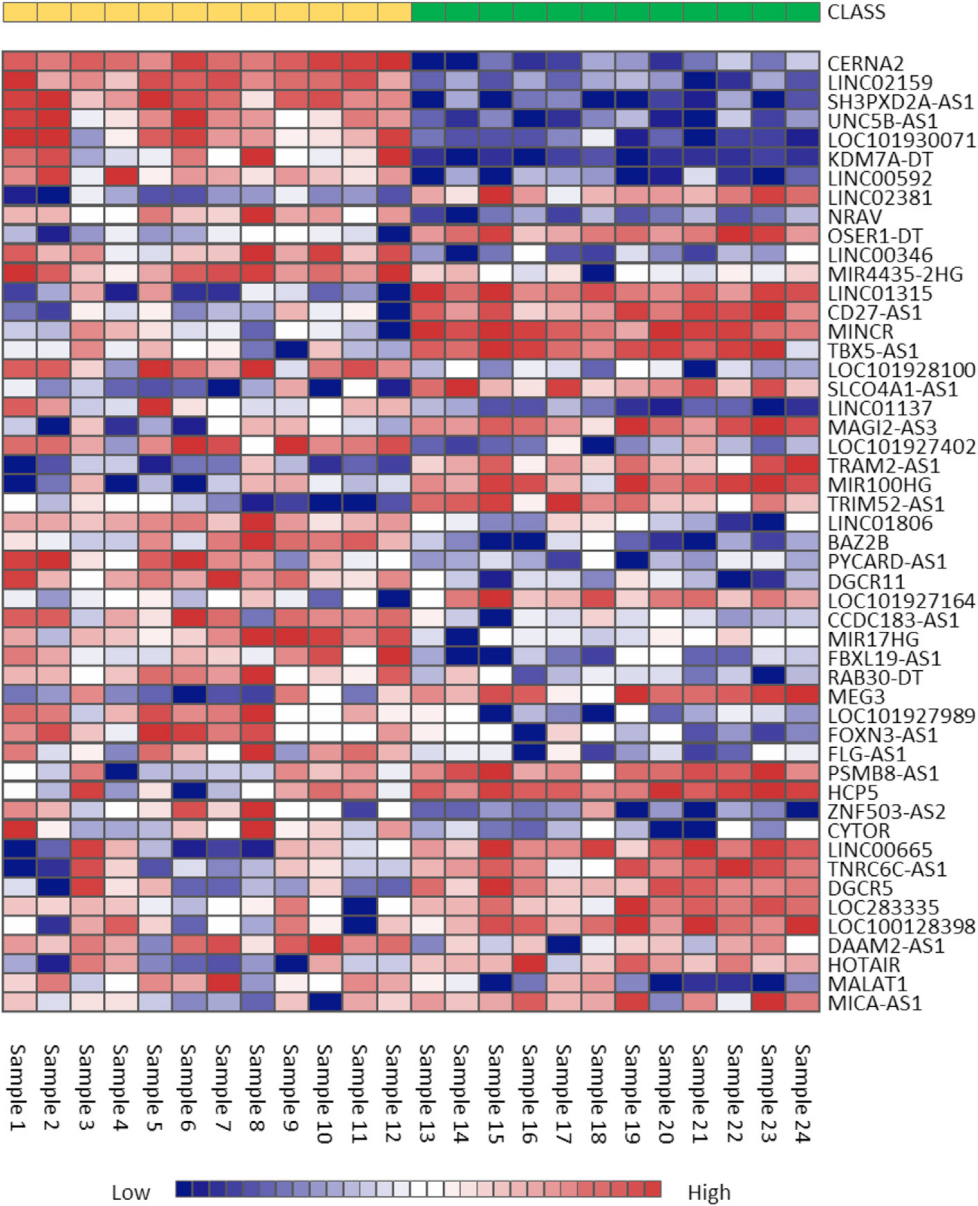
Heatmap of the most significantly differentially expressed genes. The yellow class represents warts (samples 1–12), while the green class represents normal skin (samples 13–24). *For each lncRNA, red signifies upregulated expression while blue indicates downregulated expression.*

**Figure 4:**
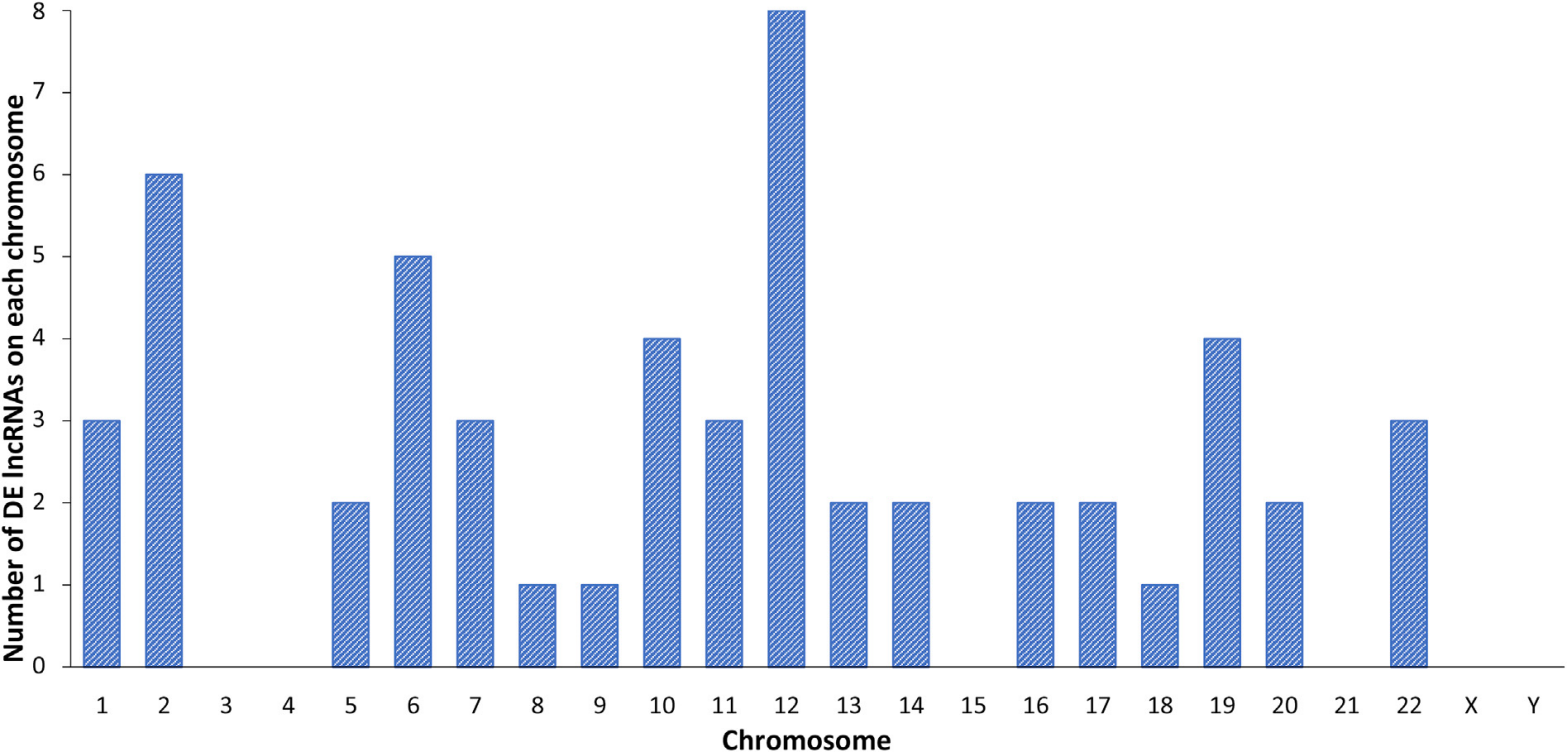
Distribution of differentially expressed (DE) lncRNAs in each chromosome.

Figure 2 depicts a volcano plot of the 229 significantly DE lncRNAs in common warts, which have an adjusted p-value of less than 0.05. When adding a significance cutoff of logFC < -1 or >1, 54 genes are found to be significantly DE, which are depicted in a clustered heatmap (Figure 3). Lastly, Figure 4 shows the distribution of DE lncRNAs on each chromosome.

### 3.2 Pathway and ontology analysis

No KEGG and Reactome pathways were found to be associated with the 229 significantly DE lncRNAs in common warts. Similarly, gene ontology (GO) term enrichment analysis did not yield any significant GO terms.

### 3.3 Gene regulatory networks

**Figure 5:**
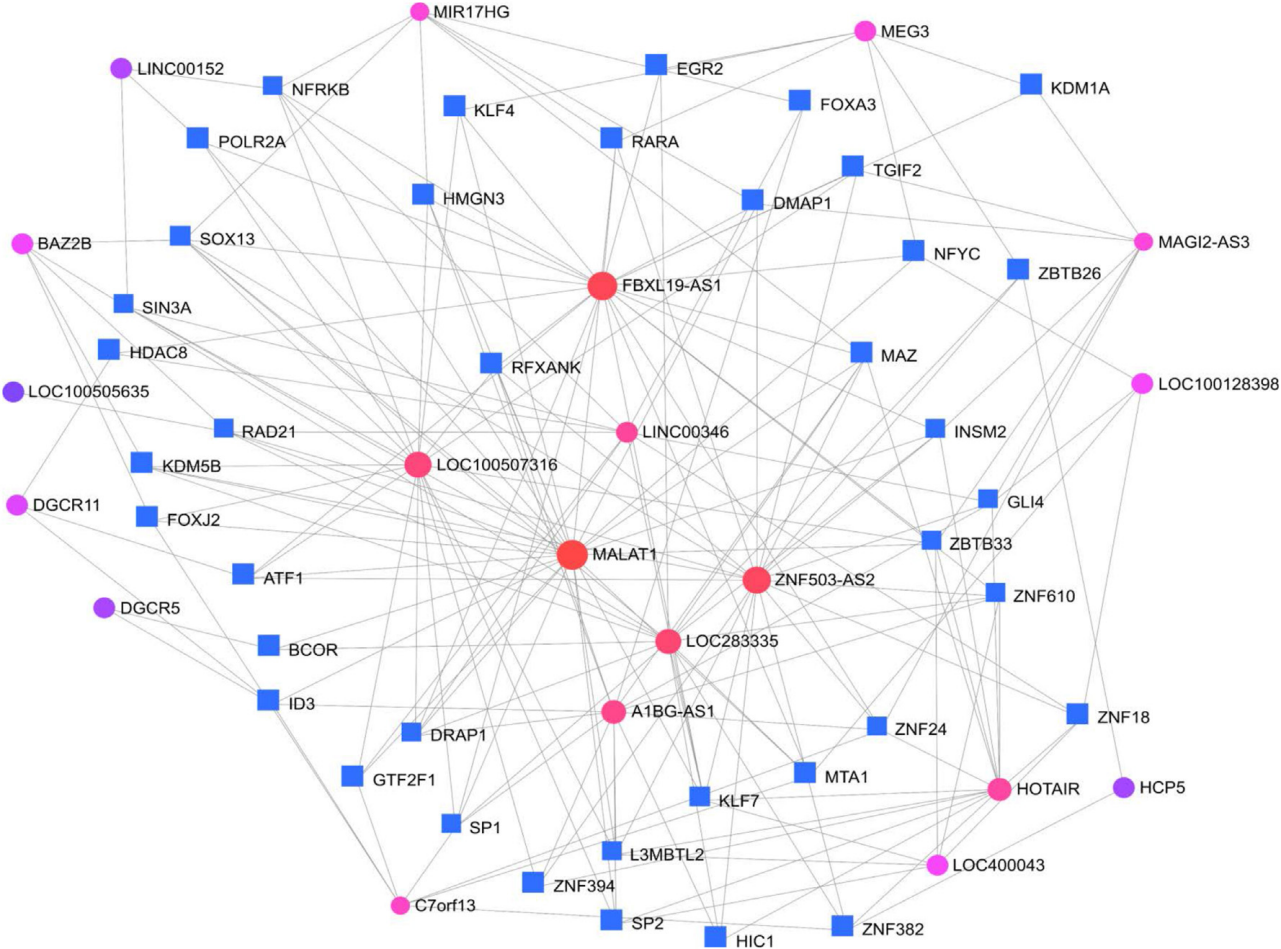
Analysis of the interaction network between transcription factors (blue squares) and lncRNAs (the colored circles) in common warts. *The size of the colored circle signifies the number of interactions.*

Figure 5 shows the analysis of the interaction network between transcription factors and lncRNAs in common warts.

### 3.4 Disease-lncRNA associations

**Figure 6:**
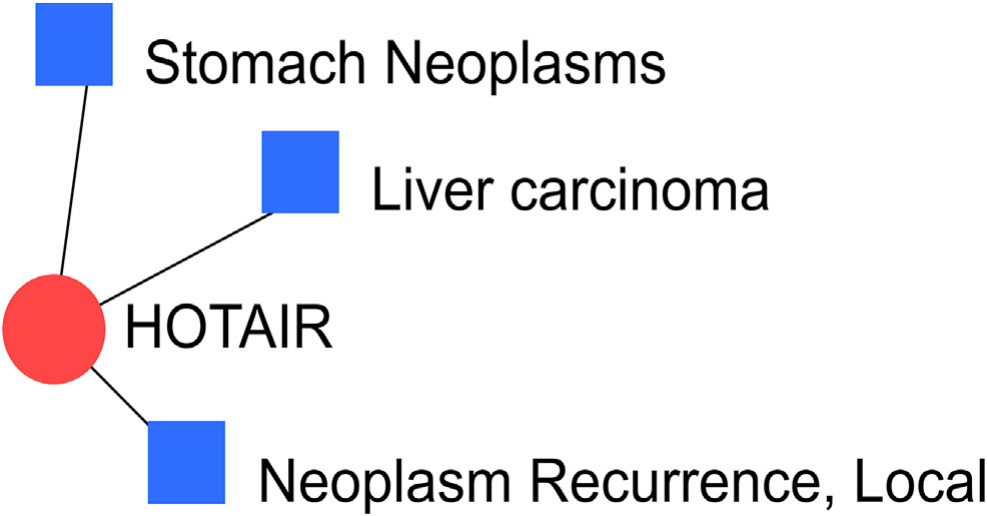
Diseases associated with the *HOTAIR* lncRNA gene.

3 diseases have been found to be associated with *HOTAIR*, while *MIR4435-2HG* is associated with lung neoplasms. Figure 6 displays the diseases associated with the *HOTAIR* lncRNA gene.

#### 3.4.1 lncRNA-miRNA interactions

**Figure 7:**
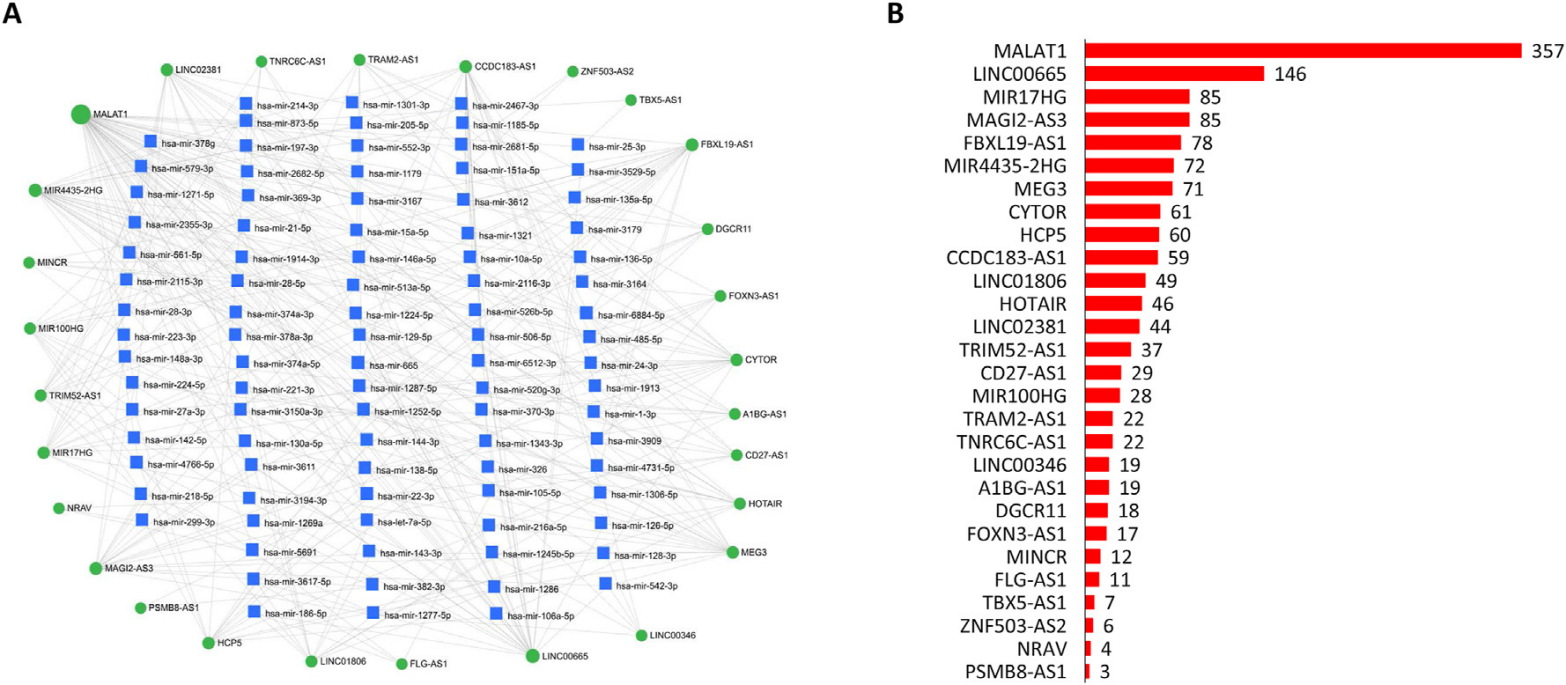
lncRNA-miRNA interaction networks showing the (A) DE lncRNAs in common warts and (B) the number of miRNA targets for each DE lncRNA. *The green color signifies lncRNAs while the blue color indicates miRNAs. The size of the node corresponds with the number of connections.*

**Figure 8:**
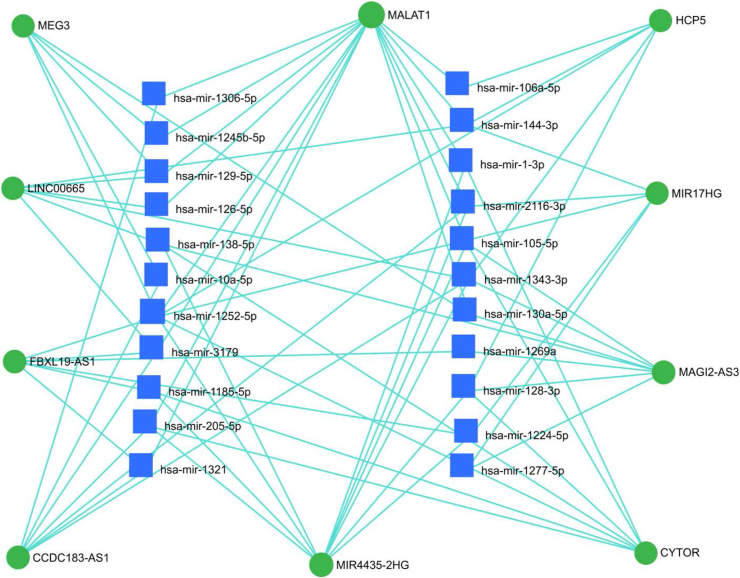
lncRNA-miRNA interaction networks showing the top 10 most DE lncRNAs in common warts. *The green color signifies lncRNAs while the blue color indicates miRNAs. The size of the node corresponds with the number of connections. Only lncRNAs with at least 49 connections with miRNAs were included.*

Out of 54 DE lncRNA, 28 DE lncRNAs were mapped to 523 miRNAs. The interaction network included 551 nodes and 1,263 edges. Due to the large number of nodes and edges, only the 28 most DE lncRNAs with the common shared miRNAs are presented in Figures 7A and 7B. Figure 8 shows the top 10 most DE lncRNAs with at least 49 connections with miRNAs on the original interaction network.

#### 3.4.2 lncRNA-miRNA interactions

**Figure 9:**
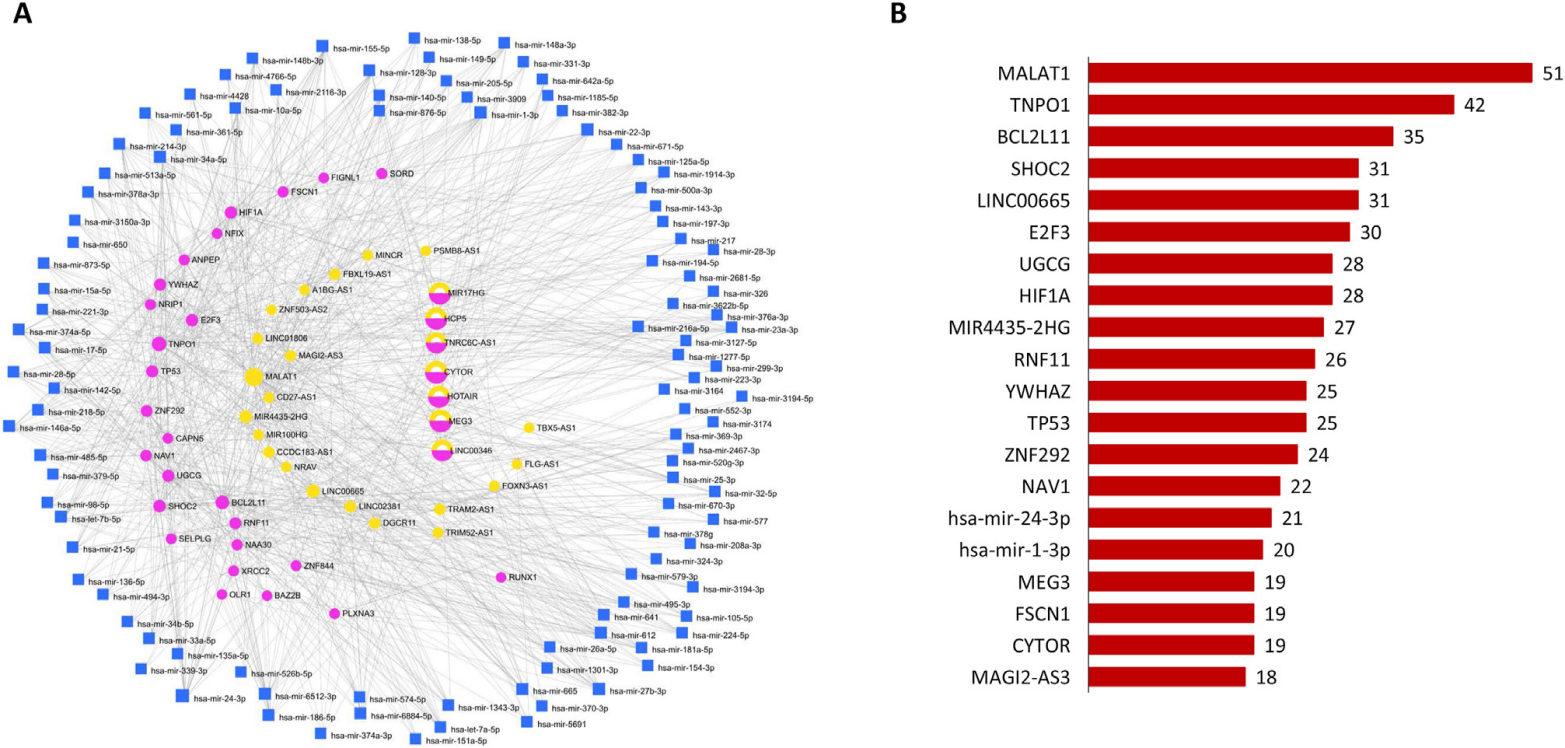
lncRNA-miRNA-gene interaction networks showing the (A) DE lncRNAs, DE miRNAs, and DE genes in common warts and (B) the number of miRNA and gene targets for each DE lncRNA. *The blue square signifies DE miRNAs, the pink circle indicates the DE genes, the yellow circle indicates DE lncRNAs while the circle with mixed yellow and pink colors represents lncRNAs which are also annotated for genes.*

The result of the analysis revealed a total of 7,137 nodes, of which 4,423 are genes, 2,686 are miRNAs, and 28 are lncRNAs. To simplify the network visualization, the 28 lncRNAs were extracted with their common shared miRNA (104 nodes) and genes (24 nodes) (Figures 9A and 9B).

### 3.5 Validation of differentially expressed lncRNAs

Merger of the most DE and DM lncRNAs in common warts revealed 21 overlapping lncRNA genes (Table 2).

**Table 2:** List of lncRNAs with significant differential expression and methylation levels in warts compared to normal skin.

Gene	Expression level			Methylation level		
	logFC	FDR	Regulation	mean.mean.diff (W-NS)	comb.p.adj.fdr	Methylation
LINC02159	-3.335691	1.55 × 10^−10^	Down	-0.136517	2.90 × 10^−05^	Hypo
OSER1-AS1	2.007927	3.24 × 10^−08^	Up	0.001901	2.85 × 10^−02^	Hyper
LINC02381	2.382596	3.66 × 10^−08^	Up	0.048269	1.93 × 10^−02^	Hyper
UNC5B-AS1	-5.36652	6.72 × 10^−08^	Down	-0.035674	4.95 × 10^−03^	Hypo
CD27-AS1	1.583693	5.02 × 10^−07^	Up	0.041279	9.22 × 10^−03^	Hyper
MINCR	1.590269	7.80 × 10^−07^	Up	-0.072536	7.99 × 10^−05^	Hypo
LINC01315	2.263108	8.33 × 10^−07^	Up	0.074438	1.98 × 10^−03^	Hyper
SLCO4A1-AS1	1.667457	1.73 × 10^−06^	Up	-0.008964	4.48 × 10^−02^	Hypo
RPARP-AS1	1.145768	2.94 × 10^−06^	Up	-0.015154	3.54 × 10^−02^	Hypo
MIR100HG	2.794321	2.48 × 10^−05^	Up	0.050978	1.24 × 10^−02^	Hyper
MIRLET7BHG	1.004021	4.17 × 10^−05^	Up	-0.01918	1.67 × 10^−02^	Hypo
BAIAP2-AS1	1.221853	1.94 × 10^−04^	Up	0.033241	4.02 × 10^−02^	Hyper
LINC01806	-1.43113	2.36 × 10^−04^	Down	0.031243	2.06 × 10^−02^	Hyper
AC005083.1	1.022727	4.23 × 10^−04^	Up	0.042764	8.59 × 10^−04^	Hyper
AC068888.1	1.452133	6.30 × 10^−04^	Up	0.08298	2.64 × 10^−04^	Hyper
MIR17HG	-1.22416	8.77 × 10^−04^	Down	-0.01407	1.80 × 10^−02^	Hypo
LINC00852	1.010049	1.01 × 10^−03^	Up	0.092531	1.13 × 10^−03^	Hyper
HOTAIR	1.398526	1.77 × 10^−03^	Up	0.071102	1.56 × 10^−02^	Hyper
FOXN3-AS1	-1.00561	1.89 × 10^−03^	Down	0.029592	3.80 × 10^−02^	Hyper
FLG-AS1	-1.23107	2.60 × 10^−03^	Down	-0.02108	4.58 × 10^−02^	Hypo
DNM3OS	1.164727	7.53 × 10^−03^	Up	0.098868	6.96 × 10^−04^	Hyper

*comb.p.adj.fdr*: FDR adjusted combined p-value of all sites; *FDR*: false discovery rate; *logFC*: base 2 logarithm of fold change; *mean.mean.diff (W-NS)*: the mean of the mean methylation levels across all sites difference between warts (W) and normal skin (NS).

## 4 Discussion

Previous evidence indicated that lncRNAs are deregulated by the high-risk HPV proteins E6 and E7 [7]. However, the role and impact of E6 and E7 proteins from low-risk HPV types remain poorly understood compared to their high-risk counterparts [11]. Here, we found that 54 lncRNAs were DE in common warts, 24 of which were upregulated and 30 of which were downregulated. The most significantly DE lncRNAs in common warts were *CERNA2*, *LINC02159*, *SH3PXD2A-AS1*, and *UNC5B-AS1.* In common warts, the most downregulated lncRNA was *UNC5B-AS1* and the most upregulated lncRNA was *MIR100HG.*

*CERNA2*, also known as *HOST2*, was the most significantly DE lncRNA in common warts and was downregulated. *CERNA2* acts as an oncogenic lncRNA, and silencing of *CERNA2* expression was found to inhibit the proliferative, migratory, and invasive capacities of breast, cervical, gastric, and osteosarcoma cancer cells [27, 28, 29, 30]. It was also suggested to increase epithelial–mesenchymal transition in hepatocellular carcinoma tissues via the JAK2-STAT3 signaling pathway [31]. Compared to normal skin, *CERNA2* was significantly upregulated in psoriatic skin and positively correlated with *STAT1*, a gene whose expression is inhibited by HPV proteins for episome maintenance and genome amplification [32, 33]. Additionally, *CERNA2* was up-regulated in HPV-positive cervical cancer cells, resulting in the down-regulation of its target microRNA let-7b [34].

*LINC02159*, also known as *LOC285629*, was DE in thyroid cancer, HER2^+^HR^−^ breast cancer, and metastatic melanoma, and it was identified as a part of lncRNA signatures in colorectal cancer as well as head and neck squamous cell carcinoma [35, 36, 37, 38, 39, 40]. *LINC02159* was classified as a low-risk gene for esophageal squamous cell carcinoma, and it is significantly associated with colorectal cancer prognosis [41].

Evidence suggests that *SH3PXD2A-AS1* acts as an oncogene in colorectal cancer and esophageal squamous cell carcinoma, with *SH3PXD2A-AS1* knockdown resulting in the inhibition of colorectal tumor growth and migration [42, 43, 44, 45]. *SH3PXD2A-AS1* was markedly upregulated in psoriatic skin, and it was identified as part of a positive feedback loop that regulated the proliferation of keratinocytes and affected the pathogenesis of psoriasis [32, 46].

*UNC5B-AS1*, also known as *UASR1*, was found to be downregulated in senescent normal oral keratinocytes, [47]. In contrast, *UNC5B-AS1* upregulation increased cell proliferation in breast, cervical, colorectal, oral squamous cell, and papillary thyroid cancers [48, 49, 50, 51, 52].

*MIR100HG* overexpression was associated with cetuximab resistance and poor prognosis in colorectal and cervical cancer patients, and it was found to promote cell proliferation in laryngeal squamous cell carcinomas, hepatocellular carcinomas, osteosarcomas, and triple-negative breast cancer [53, 54, 55, 56, 57, 58, 59, 60]. Recently, *MIR100HG* was reported to modulate the transforming growth factor beta (TGFβ) signaling pathway, the latter of which is subjugated by HPV in head and neck squamous cell carcinoma and induces epithelial-to-mesenchymal transition in keratinocytes transformed by HPV16 [61,62,63].

Our findings showed that *MALAT1* had the highest number of interactions with microRNAs and protein-coding genes that were previously found to be DE in common warts. Upregulated *MALAT1* expression was identified in high-risk HPV-positive cervical squamous tissues and cell lines, and *MALAT1* was suggested to modulate cellular apoptosis, proliferation, migration, and invasion [64, 65, 66, 67]. Previous findings have also indicated that *MALAT1* induces epithelial-mesenchymal transition in breast, cervical, colorectal, esophageal, and prostate cancer cells as well as in non-transformed cell lines, namely ARPE-19 cells, HK-2 cells, and lens epithelial cells [68, 69, 70, 71, 72, 73, 74, 75, 76]. High-risk HPV16 was reported to regulate *MALAT1* expression in cervical cancer cells, whereby *MALAT1* overexpression was dependent on the expression of the HPV16 E7 oncoprotein [77].

As highlighted above, several of the DE genes in common warts were previously reported to be dysregulated in psoriatic skin. Certain HPV types are highly prevalent in psoriatic skin, and a nationwide population-based cohort study in Taiwan indicated that there is a possible link between HPV infection and the risk of developing psoriasis [78, 79, 80]. The *STAT3* gene, which plays a critical role in psoriasis pathogenesis, is essentially activated by high-risk HPVs in both differentiated and undifferentiated keratinocytes [46, 81].

Likewise, many of the DE genes in our study previously reported to be dysregulated in colorectal cancer. Like psoriasis, HPV positivity resulted in an increased risk of colorectal carcinoma, and HPV infection has been suggested to be a co-factor in the pathogenesis of colorectal cancer [82, 83]. Small population studies have shown that colorectal infection with high-risk HPV types is common in colorectal cancer patients, but large population studies have failed to replicate this finding [84, 85, 86, 87]. In any case, the exact role that HPV plays in colorectal tumor carcinogenesis is remains to be determined.

## 5 Conclusion

Our findings indicate that HPV-induced warts impact the expression profiles of certain lncRNA genes. To the best of our knowledge, the present study is the first to explore the impact of low-risk HPV infection on lncRNA expression profiles. Nonetheless, our findings are limited by the relatively small sample size as well as the all-male patient cohort, and they must be validated in a larger cohort. Moreover, more research needs to be carried out in order to determine whether the changes in lncRNA expression were induced as part of the host's response to infection or from the HPV itself.

## Declarations

### Author contribution statement

Amneh H. Tarkhan, Mansour A. Alghamdi: Performed the experiments; Analyzed and interpreted the data; Contributed reagents, materials, analysis tools or data; Wrote the paper.

Laith N. AL-Eitan: Conceived and designed the experiments; Performed the experiments; Analyzed and interpreted the data; Contributed reagents, materials, analysis tools or data; Wrote the paper.

Rami Q. Alkhatib: Analyzed and interpreted the data; Contributed reagents, materials, analysis tools or data.

### Funding statement

This work was supported by the Deanship of Scientific Research at King Khalid University through the Small Groups Project (grant number: RGP. 1/66/43). This work was also supported by the Deanship of Research at Jordan University of Science and Technology (177/2017).

### Data availability statement

Data associated with this study has been deposited at NCBI GEO under the accession number GSE136347.

### Declaration of interests statement

The authors declare no conflict of interest.

### Additional information

No additional information is available for this paper.
